# Randomized Phase III Study (ADMYRE) of Plitidepsin in Combination with Dexamethasone vs. Dexamethasone Alone in Relapsed/Refractory Multiple Myeloma: Results for Patients Aged <75 Years

**DOI:** 10.3390/cancers17213482

**Published:** 2025-10-29

**Authors:** María Victoria Mateos, Evangelos Terpos, Sara Martínez, Carmen Kahatt, Javier Jiménez, Sonia Extremera, Javier Gómez, Vicente Alfaro, Heinz Ludwig

**Affiliations:** 1 Department of Hematology, University Hospital of Salamanca/IBSAL, 37007 Salamanca, Spain; 2 Department of Clinical Therapeutics, School of Medicine, National and Kapodistrian University of Athens, 11528 Athens, Greece; eterpos@med.uoa.gr; 3 Clinical R&D, PharmaMar, Colmenar Viejo, 28770 Madrid, Spain; smgonzalez@pharmamar.com (S.M.); ckahatt@pharmamar.com (C.K.); jjjimenez@pharmamar.com (J.J.); sextremera@pharmamar.com (S.E.); jgomez@pharmamar.com (J.G.); valfaro@pharmamar.com (V.A.); 4 Wilhelminen Cancer Research Institute, Department of Medicine, Center for Oncology, Hematology and Palliative Care, Wilhelminen Hospital, 1160 Vienna, Austria; heinz.ludwig@aon.at

**Keywords:** plitidepsin, dexamethasone, progression-free survival, overall survival, multiple myeloma

## Abstract

**Simple Summary:**

Age-related heterogeneity in efficacy has been found in several clinical trials evaluating new therapies in relapsed/refractory multiple myeloma (r/r MM). The aim of this pre-planned analysis from the ADMYRE trial was to evaluate the efficacy and safety of plitidepsin plus dexamethasone (DXM) compared to DXM alone in patients aged <75 years. The results show improved efficacy outcomes while also maintaining a similar safety profile compared to the overall ADMYRE population. These findings support the fact that this combination should be considered as another therapeutic option available for patients with extensively pretreated r/r MM.

**Abstract:**

*Background:* The phase III ADMYRE trial evaluated plitidepsin plus dexamethasone (DXM) versus DXM alone in patients with relapsed/refractory multiple myeloma (r/r MM). ADMYRE met its primary endpoint, showing a 35% reduction in the risk of progression or death. *Methods:* Results from a pre-planned subgroup of patients aged <75 years are shown here. This subgroup includes most of the patients evaluated in the ADMYRE study: 145/171 patients (84.8%) in the plitidepsin + DXM arm and 71/84 (84.5%) patients in the DXM alone arm. *Results:* Compared to the overall ADMYRE population, a higher reduction was found with plitidepsin plus DXM for the risk of progression or death in the primary endpoint: 47.7% vs. 35.0% (Hazard ratio [HR] = 0.523 vs. HR = 0.6509). Higher reduction in the risk of death was also found (28.9% vs. 20.3%; HR = 0.711 vs. HR = 0.797), with a clinically meaningful 5-month difference in median overall survival (13.0 months vs. 8.1 months; *p* = 0.0350). The safety profile of plitidepsin plus DXM in patients aged <75 years was similar to that observed in the overall population of patients treated in the ADMYRE study. The most common adverse events (all grades) related to the study treatment in patients < 75 years were fatigue (39.2% of patients), gastrointestinal (nausea, 39.2%; vomiting, 19.6%; diarrhea, 14.7%), and myalgia (14.0%). *Conclusions:* Larger differences in efficacy outcomes while maintaining a similar safety profile, together with a novel mechanism of action, suggest that this combination can be a valid option for patients with r/r MM aged <75 years.

## 1. Introduction

Plitidepsin plus dexamethasone (DXM) showed activity in a phase II clinical trial conducted in relapsed/refractory multiple myeloma (r/r MM) patients [1] and was later assessed versus DXM alone in the randomized phase III ADMYRE trial [2]. ADMYRE met its primary endpoint and showed prolongation of progression-free survival (PFS) with plitidepsin plus DXM: median PFS without disease progression (PD) confirmed by an Independent Review Committee (IRC) assessment was 2.6 months (plitidepsin plus DXM) and 1.7 months (DXM) (HR = 0.650; *p* = 0.0054). Median overall survival [OS, intention-to-treat (ITT) analysis] was 11.6 months (plitidepsin plus DXM) and 8.9 months (DXM) (HR = 0.797; *p* = 0.1261). The safety profile was tolerable and did not overlap with the toxicity observed with other agents used in MM [2]. Based on these findings, the plitidepsin plus DXM combination was approved in Australia in 2018 for the treatment of patients with r/r MM.

According to the *European Medicines Agency (EMA) Guideline on the investigation of subgroups in confirmatory clinical trials* (*Section 4.3.a, EMA/CHMP/539146/2013*), heterogeneity relates to the extent of differences within a clinical trial population in factors that are prognostic for outcome or predictive of treatment effects: the more heterogeneous the population, the more important the investigation of treatment effects in well-defined subgroups. Age-related heterogeneity in efficacy outcomes (PFS and overall survival, OS) has been found in several clinical trials evaluating new therapies in r/r MM [3,4,5]. Overall, these findings highlight the critical importance of reporting subgroup analyses by age and support the importance of evaluating subgroups for the determination of clinical benefit [6].

Seventy-five years has been used as the age cutoff point in previous trials in MM for reporting data [4,5] and has been found to be clinically relevant [7], as two thirds of patients with newly diagnosed MM are aged <75 years [8], and represents a distinct cohort with less advanced disease at diagnosis, as reflected by lower International Staging System stage, and with a lower incidence of renal failure and anemia. Results for plitidepsin plus DXM in the pre-planned subgroup of patients aged <75 years of the ADMYRE trial are shown here.

## 2. Materials and Methods

The multicenter, multinational ADMYRE study (*clinicaltrials.gov identifier: NCT01102426*) received protocol assistance by the Committee for Medicinal Products for Human Use, was approved by the Health authorities and the Independent Local Ethics Committee of each participating center (the study was conducted at 61 investigational sites from 17 countries including Australia, Austria, Belgium, Czech Republic, France, Germany, Great Britain, Greece, Italy, The Netherlands, New Zealand, Poland, Portugal, Spain, South Korea, Taiwan and the USA) and was conducted in accordance with the Declaration of Helsinki, Good Clinical Practice guidelines, and local regulations on clinical trials. Signed informed consent was obtained from all patients prior to any study-specific procedure.

The ADMYRE trial received Scientific Advice from the Committee for Medicinal Products for Human Use (CHMP) of the European Medicines Agency (EMA) prior to starting the study. Scientific Advice involved the proposed study population; the planned dose and schedule; the control arm comparator (DXM); the primary endpoint; and the statistical analyses. In this Scientific Advice, it was recognized that DXM was an active compound that had been widely used as a single agent as well as a part of combination regimens for the treatment of MM patients. A low-dose DXM regimen was chosen because, based on data from the last available publications at the time of protocol writing, DXM administered at reduced doses was associated with a better safety profile while reporting better survival [9].

The ADMYRE study design and main results have been described elsewhere [2]. Briefly, eligible subjects were patients ≥ 18 years old with r/r MM after at least three, but not more than six, prior therapeutic regimens, including at least bortezomib and lenalidomide or thalidomide; measurable disease; Eastern Cooperative Oncology Group performance status (ECOG PS) ≤ 2; life expectancy ≥ 3 months; and adequate major organ function. Patients were stratified according to their ECOG PS score (0 and 1 vs. 2) and Durie-Salmon stage (I/II vs. III) and were randomly assigned (2:1) to receive plitidepsin 5 mg/m^2^ Day (D)1,15 plus low-dose DXM 40 mg D1,8,15,22 (Arm A, *n* = 171) or low-dose DXM 40 mg D1,8,15,22 (Arm B, *n* = 84) every four weeks (q4wk). Patients in the control arm (DXM, Arm B) with documented disease progression after a minimum of eight weeks from randomization could cross over to the combination arm (Arm A).

The primary efficacy endpoint was PFS assessed according to an IRC per the International Myeloma Working Group (IMWG) criteria current at the time of study protocol design (i.e., for the evaluation of PFS, a first PD event was assigned, but two consecutive evaluations for confirming PD were not required) [10]. OS was a secondary endpoint.

Safety was evaluated in all patients who received at least one dose of the study treatment by assessment of adverse events (Aes), clinical laboratory test results, physical examinations, and vital signs.

Kaplan–Meier estimates, the unstratified log rank test, and Cox regression were used to compare the time-to-event endpoints (e.g., PFS/OS) and to calculate the risk reduction. The study was powered for the evaluation of the main endpoint (PFS) and to ascertain if a trend in OS was observed in favor of the experimental arm. Analysis of efficacy and safety in subgroups by age was preplanned in the ADMYRE Statistical Analysis Plan to characterize a potential benefit.

## 3. Results

### 3.1. Patient Characteristics

In ADMYRE, a total of 255 patients were randomized (2:1) between June 2010 and May 2015: 171 in Arm A and 84 in Arm B. The subgroup of patients aged <75 years includes most of the patients evaluated in the ADMYRE study: 145 out of the 171 patients (84.8%) in the plitidepsin plus DXM arm and 71 out of the 84 (84.5%) patients in the DXM alone arm. The baseline characteristics of patients aged <75 years were well balanced between treatment arms despite this subgroup having a non-randomized basis (Table 1).

### 3.2. Efficacy in Patients Aged <75 Years

**Figure 1:**
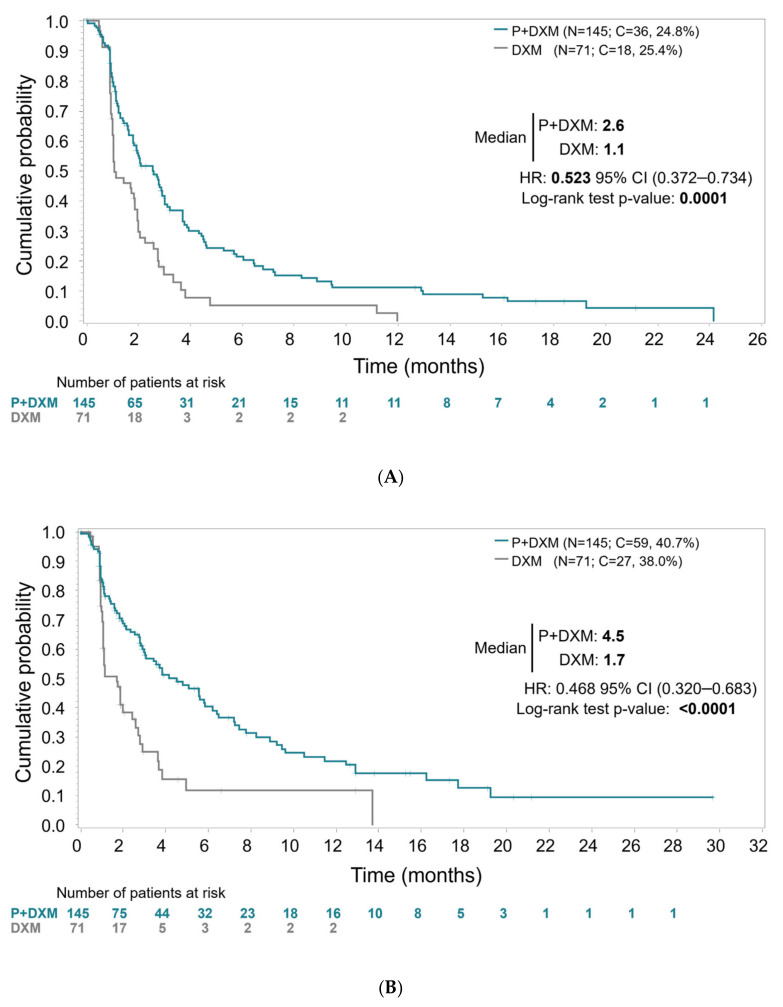
(**A**) Kaplan–Meier curve for progression-free survival without disease progression (PD) confirmation by Independent Review Committee in patients aged <75 years. (**B**) Kaplan–Meier curve for progression-free survival with PD confirmation by the investigator’s assessment in patients aged <75 years. Abbreviations: C, censored; DXM, dexamethasone; HR, hazard ratio; P, plitidepsin; N, number of patients.

The primary efficacy analysis, with blinded IRC assessment of all randomized patients performed without PD confirmation, showed statistically significant longer PFS for patients aged <75 years treated with plitidepsin plus DXM. Median PFS was 2.6 months (95% CI, 1.8–3.0 months) in Arm A (plitidepsin plus DXM) and 1.1 months (95% CI, 1.0–1.9 months) in Arm B (DXM) (log-rank *p* < 0.0001) (Figure 1). The relative risk of progression or death was reduced by 47.7% in patients treated with plitidepsin plus DXM (HR = 0.523; 95%CI, 0.372–0.734; *p* < 0.0002).

PFS analysis requiring PD confirmation by investigator’s assessment (a planned sensitivity analysis) showed median PFS of 4.5 months (95% CI, 3.0–5.8 months) in Arm A (plitidepsin plus DXM) and 1.7 months (95% CI, 1.1–2.4 months) in Arm B (DXM) (log-rank *p* < 0.0001), with a relative risk of progression or death reduced by 53.2% in patients treated with plitidepsin plus DXM (HR = 0.468, 95%CI, 0.320–0.683; *p* < 0.0001).

The median (range) cycle where the first response was obtained was Cycle 1 (Cycle 1-Cycle 11) in Arm A (plitidepsin plus DXM) and Cycle 2 (only one response was observed) in Arm B. The median (range) time on treatment of patients with response was 33.1 weeks (7.3–157.9 weeks) in Arm A (plitidepsin plus DXM) and 24.0 weeks in Arm B.

**Figure 2:**
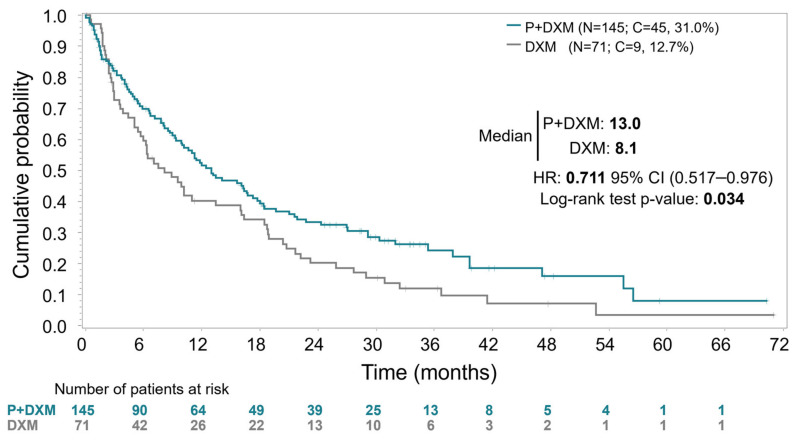
Kaplan–Meier curves for overall survival in all randomized patients aged <75 years. Abbreviations: C, censored; DXM, dexamethasone; HR, hazard ratio; P, plitidepsin; N, number of patients.

Mature final intention-to-treat OS analysis was based on 162 death events (75.0% of the 216 randomized patients aged <75 years). Median OS was 13.0 months (95% CI, 9.9–17.3 months) in Arm A (plitidepsin plus DXM) and 8.1 months (95% CI, 5.7–16.0 months) in Arm B (DXM) (log-rank *p* = 0.0340) (Figure 2). Despite the crossover in the control arm to the experimental arm of 31 patients (43.7%), the relative risk of death was reduced by 28.9% in patients treated with plitidepsin plus DXM (HR = 0.711, 95%CI, 0.517–0.976; *p* = 0.0350).

Two-stage OS analysis, which mitigates the effect of crossover, showed a more statistically significant difference in favor of Arm A (plitidepsin plus DXM). Median OS remained as 13.0 months (95% CI, 9.9–17.3 months) in Arm A (plitidepsin/DXM) and was estimated as 6.4 months (95% CI, 5.0–8.6 months) in Arm B (DXM) (log-rank *p* < 0.0001). Relative risk of death was reduced by 46.6% in patients treated with plitidepsin plus DXM (HR = 0.534, 95%CI, 0.386–0.738; *p* < 0.0001).

### 3.3. Safety in Patients Aged <75 Years

All treated patients (*n* = 143 in Arm A, plitidepsin plus DXM, and *n* = 70 in Arm B, DXM) were evaluable for safety. The median (range) of cycles received was 3 (1–39) in Arm A (plitidepsin plus DXM) and 2 (1–27) in Arm B (DXM). The median (range) time on treatment was 12.3 weeks (1.3–162.3 weeks) in Arm A (plitidepsin plus DXM) and 8.4 weeks (1.4–56.0 weeks) in Arm B. In Arm A (plitidepsin plus DXM), the most common AEs (all grades) related to the study treatment (or with unknown causality) in patients aged <75 years were nausea (39.2% of patients), fatigue (39.2%), vomiting (19.6%), diarrhea (14.7%) and myalgia (14.0%). The most common grade 3/4 treatment-related (or with unknown causality) AEs were fatigue (11.2%), myalgia (4.2%) and nausea (3.5%) (Table 2). The most common grade 3/4 hematological abnormalities were anemia (30.7%), lymphopenia (21.2%), thrombocytopenia (20.4%) and neutropenia (13.9%). Most of these hematological abnormalities were already present at baseline, as expected for patients with r/r MM. The most common grade 3/4 biochemical abnormalities were increases in creatine phosphokinase (18.0%) and alanine aminotransferase (14.7%).

In Arm B (DXM), the most common AEs (all grades) related to the study treatment (or with unknown causality) in patients aged <75 years were nausea (12.9%), insomnia (10.0%) and fatigue (8.6%). All grade 3/4 AEs occurred in one patient each (1.2%) (Table 2). The most common grade 3/4 hematological abnormalities were anemia (35.8%), thrombocytopenia (31.3%), lymphopenia (16.7%), and neutropenia (4.5%). The most common grade 3/4 biochemical abnormality was increased creatinine (6.0%).

Tolerance to treatment may also be evaluated by the time to performance status deterioration measured by ECOG PS or death, indicative of the patients’ general status. The time until ECOG PS deterioration or death was 4.7 months (95%CI, 3.0–8.4 months) for the plitidepsin plus DXM arm and 2.1 months (95%CI, 1.4–3.8 months) for the DXM arm (HR = 0.662; 95%CI, 0.472–0.928; *p* = 0.0154).

## 4. Discussion

The ADMYRE study met its primary endpoint, PFS, demonstrating benefit for the combination of plitidepsin plus DXM compared to DXM alone in r/r MM patients pretreated with at least three regimens, including bortezomib, and either lenalidomide or thalidomide [2]. The present results show a larger effect in PFS and OS of plitidepsin plus DXM compared to DXM alone in the ADMYRE trial in patients aged <75 years.

The reduction in the relative risk of progression or death in patients aged <75 years treated in the plitidepsin plus DXM arm compared to DXM alone using the most conservative method (i.e., by IRC without PD confirmation) was 47.7% (HR = 0.523; *p* < 0.0001), which is higher than that observed previously in the overall intention-to-treat population treated in ADMYRE (35.0%; HR = 0.650; *p* = 0.0054) [2]. The reduction in the relative risk of progression or death by investigator assessment with confirmation of PD (53.2%; HR = 0.468; *p* < 0.0001) is also higher than that observed in the overall population (38.9%; HR = 0.611; *p* = 0.0040) [2].

Median OS in patients aged <75 years treated with plitidepsin plus DXM was 13.0 months vs. 8.1 months with DXM alone, with a statistically significant reduction in the relative risk of death of 28.9% compared to DXM alone (HR = 0.711; *p* = 0.0350). These values were higher than the median of 11.6 months and the risk reduction in death of 20.3% (HR = 0.797; *p* = 0.1261) found in the overall population of randomized patients treated with plitidepsin plus DXM in ADMYRE [2]. The two-stage OS analysis, which mitigates the effect of crossover, also showed a higher reduction in the relative risk of death (46.6% vs. 33.3%; HR = 0.534 vs. HR = 0.667) compared to the overall ADMYRE population.

A new therapy with a novel mechanism of action that shows a PFS benefit along with a likelihood of survival benefit in a largely treatment-refractory population is considered a clinically meaningful contribution to the r/r MM therapeutic armamentarium. eEF1A2, a supposed oncogene that is overexpressed in MM, was identified as the primary target for plitidepsin [11,12]. Of note, all recently introduced new anti-MM drugs have mechanisms of activity not targeting eEF1A2.

The safety profile of plitidepsin plus DXM in patients aged <75 years was similar to that observed in the overall population of patients treated in the ADMYRE study, mainly consisting of transient laboratory abnormalities controlled by dose adjustment [2]. In this subgroup of patients aged <75 years, plitidepsin plus DXM also showed a low incidence of toxicities common with available agents used in the treatment of r/r MM (e.g., neurotoxicity, neutropenia and infections, thrombocytopenia and bleeding, or cardiac events).

## 5. Conclusions

In summary, compared to the overall population treated in the ADMYRE study, the efficacy of the combination of plitidepsin and DXM in the patient population subgroup aged <75 years was larger and characterized by a higher reduction in the risk of progression or death at the primary endpoint, PFS by IRC (47.7% vs. 35.0%); a higher reduction in the risk of progression or death at the secondary endpoint, PFS by IA with PD confirmation (53.2% vs. 38.9%); and a higher reduction in the risk of death at the secondary endpoint OS (28.9% vs. 20.3%). A difference in the median OS of about 5 months (*p* = 0.0350) is considered clinically meaningful in this heavily pretreated patient population. The combination of plitidepsin and DXM has shown anti-myeloma activity, with prolonged PFS and OS. Its distinct safety profile, in addition to its novel mechanism of action, likely provides plitidepsin with a specific place among the available options for haemato-oncologists treating r/r MM and could provide a new therapeutic option for patients aged <75 years with r/r MM. Although innovative therapies such as BCMA-directed CAR-T cells and bispecific antibodies have changed the management of relapsed disease [13], MM remains an incurable disease in which most patients continue to experience successive relapses. In this setting, drugs such as melflufen and selinexor have been approved for use in later-line r/r MM; however, some patients may not be candidates for these treatments due to their safety profile or because they have developed resistance to the main drug classes. In this context, plitidepsin plus DXM, although its efficacy appears to be more modest compared with more recent therapeutic innovations, could represent an available therapeutic alternative. Its distinct mechanism of action, targeting eEF1A2, suggests its potential for activity in patients refractory to immunomodulatory agents, proteasome inhibitors, or monoclonal antibodies, thus offering a potential option in settings with unmet medical needs.

## Figures and Tables

**Table 1:** Patient baseline characteristics (ADMYRE; patients aged <75 years).

		Arm A (Plitidepsin Plus DXM) (*n* = 145)		Arm B (DXM) (*n* = 71)		*p*-Value ^a^
		n	%	n	%	
Gender	Male	79	54.5	33	46.5	0.3109
	Female	66	45.5	38	53.5	
Age (years)	Median (range)	62 (36–74)		64 (42–73)		0.6204
ECOG PS at baseline	0	65	44.8	28	39.4	0.7708
	1	59	40.7	34	47.9	
	≥2	21 ^b^	14.5	9	12.7	
BSA (m^2^)	Median (range)	1.9 (1.3–2.5)		1.8 (1.3–2.5)		0.1085
ISS stage at baseline	I	61	42.1	33	46.5	0.4778
	II	34	23.4	15	21.1	
	III	17	11.7	12	16.9	
	NA	33	22.8	11	15.5	
Durie-Salmon stage at diagnosis	I	16	11.1	7	10.0	1.0000
	II	39	27.1	19	27.1	
	III	89	61.8	44	62.9	
Hemoglobin (g/dL)	Median (range)	10.3 (7.0–14.6)		10.0 (7.4–14.6)		0.0662
Creatinine (g/dL)	Median (range)	1 (0.5–5)		1 (0.5–2.9)		0.6646
Calcium (mg/dL)	Median (range)	9.5 (7.5–14.1)		9.5 (7.8–14.1)		0.5822
LDH (× ULN) ^c^	≤1 × ULN	101	70.6	46	65.7	0.5286
	>1 × ULN	42	29.4	24	34.3	
Number of lesions	Median (range)	1 (0–12)		1 (0–10)		0.7756
	<4 lesions	101	69.7	50	70.4	1.0000
	≥4 lesions	44	30.3	21	29.6	
Lytic lesions	Yes	98	67.6	48	67.6	1.0000
Plasmacytomas	Yes	11	7.6	10	14.1	0.1463
Genetic risk	High	39	26.9	18	25.4	0.8348
	Standard	41	28.3	18	25.4	
	NA	65	44.8	35	49.3	
Time from diagnosis to treatment	≤6 years	74	51.0	34	47.9	0.7722
	>6 years	71	49.0	37	52.1	
Time from last PD to treatment (months)	Median (range)	71.5 (0.1–217.7)		74.3 (19.5–178.9)		0.3117
Prior lines	Median (range)	4 (2–6)		4 (3–7) ^d^		0.2546
Stem cell transplantation	Yes	106	73.1	55	77.5	0.5119
Autologous	Yes	106	73.1	54	76.1	
Allogeneic	Yes	6	4.1	5	7.0	
Disease status with respect to last prior therapy ^e,f^	Refractory	59	40.7	33	46.5	0.5474
	r/r	48	33.1	19	26.8	
	Relapsed	29	20.0	12	16.9	
	Unknown	9	6.2	7	9.9	
Last prior bortezomib	r/r	36	25.2	20	28.2	0.9278
Last prior thalidomide-lenalidomide	r/r	54	37.2	27	38.0	1.0000
Last prior IMiD	r/r	54	37.2	23	32.4	0.8375
Last prior PI	r/r	41	28.7	21	29.6	1.0000

Data shown are randomized patients; n (%) or median (range). ^a^ Fisher’s exact test (categorical variables); Mann–Whitney-Wilcoxon (continuous variables). ^b^ One patient in Arm A had ECOG PS = 1 at randomization but ECOG PS = 3 before starting the study treatment. ^c^ Data not available in two (Arm A) and one patient (Arm B). ^d^ One patient in Arm B received seven prior lines; this was considered a protocol deviation. ^e^ Based on Anderson et al. [10]. ^f^ Ten patients had previously received monoclonal antibodies (daratumumab and elotuzumab): six in Arm A and four in Arm B. DXM, dexamethasone; ECOG PS, Eastern Cooperative Oncology group performance status; IMiD, immunomodulatory drugs; ISS, International Staging System; NA, not available; PD, disease progression; PI, proteasome inhibitor; r/r, relapsed/refractory; ULN, upper limit of normal.

**Table 2:** Most common laboratory abnormalities (regardless of relationship) and treatment-related adverse events in patients aged <75 years (≥10% of all treated patients).

	Arm A (Plitidepsin Plus DXM) (*n* = 143)		Arm B (DXM) (*n* = 70)	
	NCI-CTCAE Grade		NCI-CTCAE Grade	
	≥1	≥3	≥1	≥3
	n (%)	n (%)	n (%)	n (%)
Hematological abnormalities (regardless of relationship) ^a,b^				
Anemia	135 (98.5)	42 (30.7)	66 (98.5)	24 (35.8)
Lymphopenia	94 (68.6)	29 (21.2)	44 (66.7)	11 (16.7)
Thrombocytopenia	80 (58.4)	28 (20.4)	46 (68.7)	21 (31.3)
Leukopenia	68 (49.6)	9 (6.6)	33 (49.3)	1 (1.5)
Neutropenia	62 (45.3)	19 (13.9)	29 (43.9)	3 (4.5)
Lymphocytosis	12 (8.8)	3 (2.2)	1 (1.5)	.
Biochemical abnormalities (regardless of relationship) ^a,b^				
ALT increased	116 (85.3)	20 (14.7)	12 (17.9)	.
Creatinine increased	116 (84.7)	3 (2.2)	58 (86.6)	4 (6.0)
AST increased	86 (64.2)	11 (8.2)	15 (22.7)	.
CPK increased	54 (40.6)	24 (18.0)	3 (5.2)	.
AP increased	41 (30.1)	3 (2.2)	8 (12.3)	.
Bilirubin increased	16 (11.8)	3 (2.2)	5 (7.5)	.
Adverse events (treatment-related or with unknown relationship) ^b^				
Nausea	56 (39.2)	5 (3.5)	9 (12.9)	1 (1.4)
Fatigue	56 (39.2)	16 (11.2)	6 (8.6)	1 (1.4)
Vomiting	28 (19.6)	3 (2.1)	2 (2.9)	1 (1.4)
Diarrhea	21 (14.7)	2 (1.4)	2 (2.9)	.
Myalgia	20 (14.0)	6 (4.2)	2 (2.9)	.
Decreased appetite	19 (13.3)	1 (0.7)	2 (2.9)	.
Edema peripheral	17 (11.9)	1 (0.7)	1 (1.4)	.
Insomnia	10 (7.0)	.	7 (10.0)	.

The data shown are treated patients with an event. Events that occurred after the crossover were excluded from this table. ^a^ Percentages based on patients with available laboratory data. ^b^ Ordered by frequency (all grades). AP, alkaline phosphatase; ALT, alanine aminotransferase; AST, aspartate aminotransferase; CPK, creatine phosphokinase; DXM, dexamethasone; NCI-CTCAE, National Cancer Institute Common Terminology Criteria for Adverse Events.

## Data Availability

The original contributions presented in the study have been included in the article; further inquiries can be directly addressed to the corresponding author.

## Abbreviations

The following abbreviations are used in this manuscript:

AEs	Adverse Events
Day	D
DXM	Dexamethasone
ECOG PS	Eastern Cooperative Oncology Group Performance Status
EMA	European Medicines Agency
IMWG	International Myeloma Working Group
IRC	Independent Review Committee
OS	Overall Survival
PD	Disease Progression
PFS	Progression-Free Survival
r/r MM	Relapsed/Refractory Multiple Myeloma
